# Controllable Construction of Temperature-Sensitive Supramolecular Hydrogel Based on Cellulose and Cyclodextrin

**DOI:** 10.3390/polym14183801

**Published:** 2022-09-11

**Authors:** Jiayin Wu, Qilin Lu, Hanchen Wang, Beili Lu, Biao Huang

**Affiliations:** 1 Fujian Key Laboratory of Novel Functional Textile Fibers and Materials, Minjiang University, Fuzhou 350108, China; 2 College of Material Engineering, Fujian Agriculture and Forestry University, Fuzhou 350002, China

**Keywords:** supramolecular hydrogel, thermosensitivity, cyclodextrin, host–guest inclusion, grafted cellulose

## Abstract

In temperature sensitive hydrogels, the swelling degree or light transmittance of the gel itself changes with variations in ambient temperature, prompting its wide application in controlled drug release, tissue engineering, and material separation. Considering the amphiphilic structure of β-cyclodextrin (β-CD), a cellulose-based supramolecular hydrogel with superior temperature sensitivity was synthesized based on a combination of cellulose and β-CD as well as the host–guest interaction between β-CD and polypropylene glycol (PPG). In the one-pot tandem reaction process, chemical grafting of β-CD on cellulose and the inclusion complexation of β-CD with PPG were performed simultaneously in a NaOH/urea/water system. The obtained supramolecular hydrogel had a lower critical solution temperature (LCST) of 34 °C. There existed covalent bonding between the cellulose and β-CD, host–guest complexation between the β-CD and PPG, and hydrogen bonding and hydrophobic interactions between the components in the network structure of the supramolecular hydrogel. The combination of various covalent and non-covalent bonds endowed the resulting supramolecular hydrogel with good internal network structure stability and thermal stability, as well as sensitive temperature responsiveness within a certain range—implying its potential as a smart material in the fields of medicine, biology, and textiles. This work is expected to bring new strategies for the fabrication of cellulose-based thermosensitive materials, benefitting the high-value utilization of cellulose.

## 1. Introduction

Thermosensitive hydrogels can change their swelling rate or light transmittance in response to changing ambient temperatures. Thermosensitive hydrogels are widely used in the fields of controlled drug release, tissue engineering, material separation, and biomedical materials precisely because of the nature of this phase transition with temperature changes [1,2,3,4]. Considering their differences in temperature responsiveness, thermosensitive hydrogels are divided into two types: positive thermosensitive hydrogel and negative thermosensitive hydrogel. Above the upper critical solution temperature (UCST), positive thermosensitive hydrogels exist in a transparent or swollen state, while they transition to be opaque or shrink below the UCST. On the contrary, negative thermosensitive hydrogels are opaque or contracted above the lower critical solution temperature (LCST) and exist in a transparent or swelling state below the LCST. At present, the synthesis of thermosensitive hydrogels is mainly based on polyethylene oxide, polyvinyl alcohol, polyacrylic acid, polyacrylamide, and other compounds [5,6,7]. Among these, poly (N-isopropyl acrylamide; PNIPAM) is the most widely studied due to its LCST being close to body temperature and its sensitive temperature responsiveness. Research focusing on thermosensitive copolymers aims at regulating the critical solution temperature and endowing temperature-sensitive gels with excellent properties such as dual temperature-sensitivity and self-healing [8,9]. The application of the mentioned polymers and copolymers has been limited owing to their difficult degradability and low biocompatibility. Therefore, renewable, biocompatible, and environmentally friendly alternative materials have been sought in recent studies, such as polylactic acid [10] and polysaccharides [11]. Chitosan is one of the most commonly used polysaccharides in the field of thermosensitive gels, while low mechanical strength restricts its application [12,13]. Nevertheless, there are few studies on natural polymers as raw materials in this regard.

Hydrogels based on natural polymers have application prospects in biomedicine, cosmetics, food packaging, etc., owing to their remarkable biocompatibility and degradability [14,15,16,17]. As a renewable natural polymer with abundant sources, cellulose has attracted people’s attention. The involvement of cellulose can adjust the critical solution temperature and enhance the mechanical properties of thermosensitive gels [18,19]. Pure cellulose hydrogel is constructed by the bonding between hydroxyl groups of cellulose and water molecules. The intermolecular and intramolecular hydrogen bonds of cellulose are destroyed by dissolving, and then are recombined to form the hydrogel network structure. Modification and compounding with other organic polymers are imperative for cellulose to form cellulose-based composite hydrogels with special properties that can overcome the defects of the poor mechanical strength and toughness of pure cellulose hydrogels.

Cyclodextrins (CDs) consist of six, seven, or eight D-glucopyranose units linked by glycosidic bonds, corresponding to α-, β-, and γ-cyclodextrin, respectively. They are cyclic biomass polysaccharides with a conical molecular structure. The primary hydroxyl groups are located at the narrower end of the truncated cone structure, while the secondary hydroxyl groups are at the broader end. The interior of cyclodextrins is composed of H and O atoms for the hydrophobic cavity, and the exterior is hydrophilic by means of hydroxyl groups, presenting as a local chiral space [20]. The peculiar amphiphilic structure of cyclodextrins allows the inclusion of different kinds of guest compounds, such as macromolecular polymers and hydrophobic organic and hydrophobic small inorganic molecules, to form cyclodextrin inclusion complexes [21,22]. Compared with α- and γ-cyclodextrin, β-cyclodextrin has a cavity of a moderate size, which can encapsulate most of the substances that are to be solubilized. Additionally, the production process is simple and low-cost. However, there are some limitations in cyclodextrin’s water solubility that have attracted extensive interest [23]. Cyclodextrin inclusion complexes are typical supramolecular compounds with distinct physical and chemical properties due to their non-covalent bonds, which have extensive prospects in molecular switches, molecular self-assembly, stimuli-responsive organic optoelectronic materials, and intelligent materials [24,25,26].

**Figure 1:**
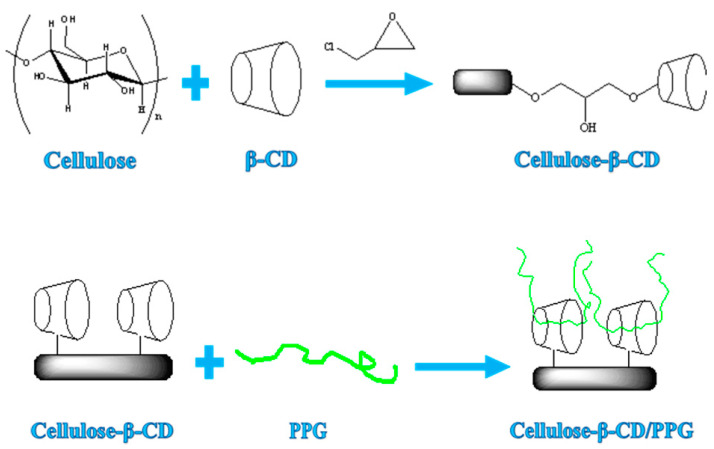
Schematic illustration of the formation of a supramolecular composite hydrogel based on cellulose, β-CD, and PPG.

Hydrogels based on covalent bonds usually require the presence of chemical cross-linking agents. Most of them have a certain level of toxicity and complex reaction processes, which restricts their use in food, drug carriers, and other applications. However, supramolecular hydrogels based on non-covalent bonds—such as host–guest complexation—can overcome these defects. Herein, cellulose-derived hydrogels were prepared by homogeneously grafting β-cyclodextrin while dissolving cellulose in a NaOH/urea/water system. Based on the amphiphilic structure of cyclodextrin, a temperature-sensitive cellulose-based supramolecular hydrogel was constructed by means of hydrogen bonding and cyclodextrin inclusion complexation (Figure 1).

## 2. Experimental

### 2.1. Materials

Bleached eucalyptus kraft pulp (BEKP) was provided by Naping Paper Co., Ltd. (Fujian, China) and the α-cellulose content was greater than 94% according to the supplier. Sodium hydroxide, urea, epichlorohydrin, and polypropylene glycol (PPG, molecular mass of 1000) were purchased by the Shanghai Aladdin Biochemical Technology Co., Ltd., (Shanghai, China). All reagents were used as received.

### 2.2. Preparation of a Cellulose-Based Supramolecular Hydrogel

First, 7 g of sodium hydroxide and 12 g urea were dissolved in 81 g of deionized water, and the mixture was frozen at −23 °C for 12 h. When the frozen mixture was thawed to an ice-water mixed state, 4 g of BEKP was added to the mixture and stirred thoroughly to obtain a transparent cellulose solution. A certain amount of β-cyclodextrin (β-CD) and epichlorohydrin were added to the cellulose solution. After stirring at 30 °C for 4 h, the cellulose mixed solution was frozen at −23 °C for 24 h to form a hydrogel. The hydrogel was repeatedly washed with deionized water to remove the lye and unreacted epichlorohydrin, and to obtain a β-CD grafted cellulose hydrogel (CH-g-β-CD). After impregnation with PPG, the CH-g-β-CD hydrogel was frozen at −23 °C for 12 h and then thawed at room temperature for 3 h to obtain a cellulose-based supramolecular hydrogel (CH-g-β-CD/PPG).

### 2.3. Characterization

After freeze-drying, the hydrogel was ground into powder and characterized by Fourier transform infrared (FTIR) and ^13^C Nuclear magnetic resonance (^13^C NMR) spectroscopy. FTIR spectra were obtained with a Nicolet 380 FTIR spectrometer (Thermo electron Instruments Co., Ltd., Waltham, MA, USA) in wavenumbers ranging from 4000 cm^−1^ to 400 cm^−1^ and a resolution of 4 cm^−1^. The ^13^C NMR analyses were carried out with an AVANCE III 400 ^13^C NMR (Bruker Corporation, Fällanden, Switzerland) at a magic angle spinning rate of 6 kHz and a proton resonance frequency of 75.5 MHz the surface and cross section morphology of the hydrogels were analyzed with a SU8010 FESEM (Hitachi, Ltd., Tokyo, Japan) at an accelerating voltage of 5 kV. The thermal stability was characterized by a thermogravimetric analyzer (NETZSCH STA 449 F3 Jupiter^®^, Hamburg, Germany) under heating from 25 °C to 600 °C at 10 °C min^−1^, with a flow of N_2_ as the protecting gas.

### 2.4. Rheology

The rheology properties of the cellulose-based hydrogels were tested using a HAAKE MARS III rheometer (Thermo Fisher Scientific, Waltham, MA, USA) with a circular shape of 2.5 mm thickness and 60 mm diameter. The supramolecular hydrogels were first subjected to a strain scan to determine the range of the linear viscoelastic behavior of the samples. Then, the strain scan parameters were set at a frequency of 1 Hz, the strain ranged from 0.01% to 100%, and the temperature was set at 25 °C in a logarithmic point pattern. The storage modulus of the supramolecular hydrogel was determined by frequency scanning under the linear viscoelastic behavior of the sample. The frequency scan parameters were set to a scan range of 0.01–10 Hz at 25 °C in logarithmic point pattern. The temperature dependence of the supramolecular hydrogels was analyzed by temperature scanning under the linear viscoelastic behavior of the samples. The temperature scan parameters were set to 1 Hz, a temperature range of 23–80 °C, and a heating rate of 2 °C min^−1^ in a logarithmic point pattern.

### 2.5. Temperature Sensitivity

The swelling properties of cellulose-based supramolecular hydrogels at different temperatures were investigated by the gravimetric method. The mass of the dried supramolecular hydrogel was recorded as *W_f_*, and it was placed in deionized water at 20–40 °C for a certain period. The samples were weighed after wiping off the residual water on the surface of the gel with filter paper, at which point the gel reached its swelling equilibrium, and the mass was recorded as *W_e_*. The equilibrium swelling rate (*SR_e_*) of the supramolecular hydrogels was calculated according to the following equation:

(1) $$SR_{e}=\frac{\left(W_{e}-W_{f}\right)}{W_{f}}\times 100\%$$

where *W_f_* is the dry weight of the hydrogel; *W_e_* is the weight of the hydrogel at equilibrium.

The phase transition behavior of the supramolecular hydrogels was reflected by the transmittance. The UV-Vis spectrophotometer was used to test the trend of the transmission of the supramolecular hydrogels at 480 nm with temperature changes in the range of 20–40 °C.

## 3. Result and Discussion

### 3.1. ^13^C NMR Analysis

**Figure 2:**
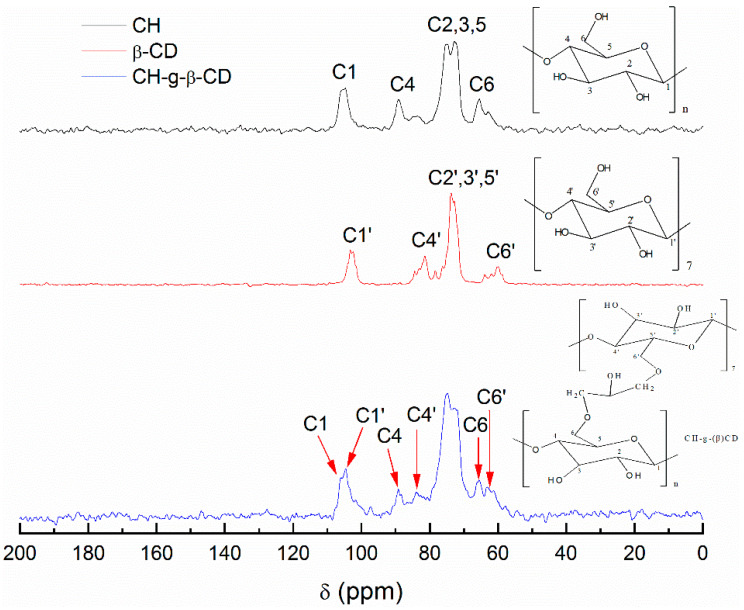
^13^C NMR spectra of the cellulose hydrogel (CH), β-CD, and CH-g-β-CD hydrogel.

Figure 2 shows the ^13^C NMR spectra of the cellulose hydrogel, β-CD, and CH-g-β-CD hydrogel. The cellulose hydrogel displayed typical signals from cellulose at 105.4 ppm, 89.1 ppm, 75.3 ppm, and 65.4 ppm—assigned to the resonance absorption of C1, C4, C2,3,5, and C6, respectively [27,28]. The chemical shifts of C2′,3′,5′ from the β-CD were the same as those from cellulose due to their identical glucose units, whereas the resonance absorptions at C1′ (103.2 ppm), C4′ (81.4 ppm), and C6′ (63.8 ppm) were different from those of the cellulose due to the diversity in the number of glucose units and their molecular conformation [29]. In the spectrum of the CH-g-β-CD hydrogel, the resonance absorption peaks from the cellulose and β-CD appeared at 105.4 ppm, 103.2 ppm, 89.1 ppm, 81.4 ppm, 65.4 ppm, and 63.8 ppm, while resolution loss occurred at 89.1 ppm and 65.4 ppm. This indicated that the chemical grafting between the cellulose and β-CD was realized by covalent bonding, which weakened the movement of the carbon atoms in the glucose units.

### 3.2. FTIR

**Figure 3:**
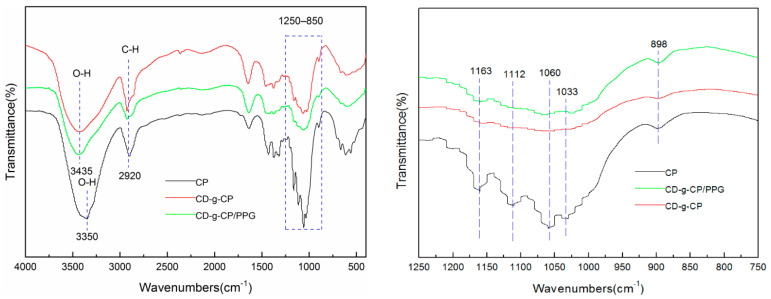
FTIR spectra of the cellulose (CP), CH-g-β-CD hydrogel, and CH-g-β-CD/PPG hydrogel.

As illustrated in Figure 3, the FTIR spectra of the CH-g-β-CD hydrogel and CH-g-β-CD/PPG hydrogel were similar to that of the cellulose, indicating that the molecular structure of the cellulose was not fundamentally damaged after chemical grafting. The strong absorption peaks near 3435 cm^−1^ from the CH-g-β-CD hydrogel and CH-g-β-CD/PPG hydrogel were assigned to the O-H stretching vibration absorption of hydroxyl groups, which shifted to a higher wavenumber; the peak shape also became narrower compared to that of cellulose (3350 cm^−1^). These changes suggest that the grafting reaction results in a change in the hydrogen bonding structure of cellulose and a weakening in intermolecular hydrogen bonding [30]. The peaks at 2920, 1638, and 1432 cm^−1^, respectively, were attributed to the vibrations of C-H, H-O-H, and C-H in the cellulose [31]. The characteristic peaks of C-O stretching vibrations (1033 cm^−1^) and β-glycosidic bonds (898 cm^−1^) were the result of the vibrational absorption of C1 from cellulose [32]. Three peaks were observed for cellulose at 1163, 1112, and 1060 cm^−1^, respectively, corresponding to the stretching vibrations of a C-C skeleton, glucose ring, and C-O attached to a hydroxyl [33]. After grafting the β-CD, the peak intensity of these three peaks decreased significantly, with a broadening at 1033 cm^−1^ for the CH-g-β-CD hydrogel and CH-g-β-CD/PPG hydrogel—indicating the formation of a covalent bond between the cyclodextrin and cellulose [34]. Compared with the CH-g-β-CD hydrogel, the increase in the peak intensity of the CH-g-β-CD/PPG hydrogel at 1033 and 898 cm^−1^ was mainly caused by C-O stretching vibrations from cyclodextrin-inclusive PPG chains, which confirmed the existence of the host–guest interaction between the β-CD and PPG in the supramolecular hydrogels [35].

### 3.3. Morphology

**Figure 4:**
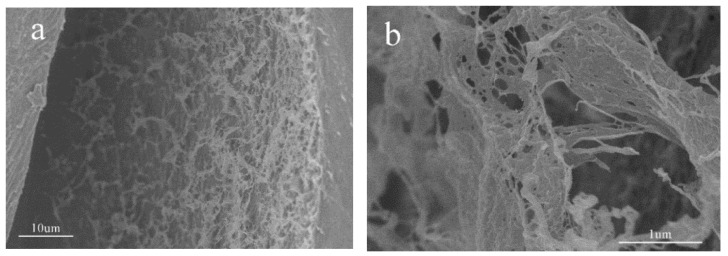

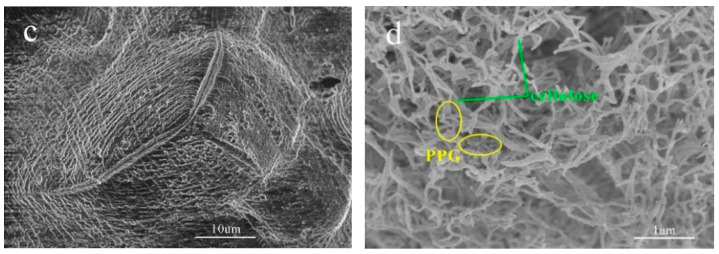
SEM images of the CH-g-β-CD hydrogel (**a**,**b**), and CH-g-β-CD/PPG hydrogel (**c**,**d**).

The cross-section morphology of the CH-g-β-CD hydrogel and CH-g-β-CD/PPG hydrogel were compared using the SEM images seen in Figure 4. It can be seen that both of them showed a developed pore structure and a connected network-like internal structure. After the inclusion of the PPG, the cellulose molecular chains and PPG chains in the network structure of the CH-g-β-CD/PPG hydrogel were tightly entangled together and formed relatively regular pore sizes (Figure 4c,d), which confirmed the orderly aggregation and good blending compatibility between cellulose and PPG. Cyclodextrin-inclusive PPG chains aggregated with each other and formed microcrystalline regions in the CH-g-β-CD/PPG hydrogel through hydrogen bonding between β-CD molecules. These microcrystalline regions served as physical cross-linking points to form a three-dimensional network structure with the uncoated PPG and cellulose molecular chains [36]. There existed hydrogen bonding and hydrophobic interactions among the β-CD, PPG, and cellulose in the network structure of the CH-g-β-CD/PPG hydrogel. These non-covalent interactions strengthened the compatibility of the components in the supramolecular hydrogel, resulting in a dense network structure.

### 3.4. Rheological Analysis

**Figure 5:**
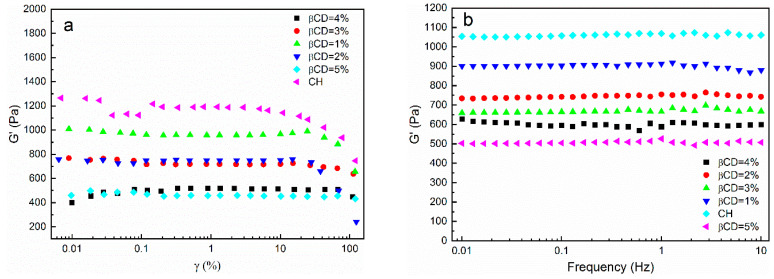

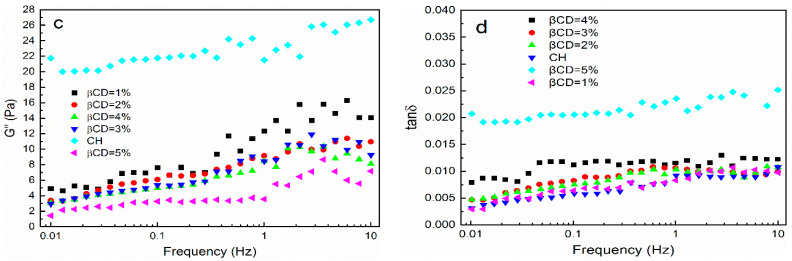
Rheological behavior of CH-g-β-CD hydrogels with different β-CD contents: (**a**) storage modulus vs. strain; (**b**) storage modulus vs. frequency; (**c**) loss modulus vs. frequency; (**d**) tanδ vs. frequency.

When the strain amplitude was less than 1%, the storage modulus curves of the hydrogels with different β-CD contents remained horizontal (Figure 5a), which suggested that the G′ was independent of the strain and that the hydrogels exhibited linear behavior. When the strain amplitude was higher than 1%, the storage modulus gradually decreased as the strain amplitude increased, and the samples exhibited non-linear behavior. Therefore, the strain amplitude should be set at 1% during the frequency scan test. As shown in Figure 5b, the storage modulus curves of the hydrogels with different β-CD contents remained flat with increasing frequencies, indicating that the CH-g-β-CD hydrogel had a certain structural stability—maintained by the hydrogen bonding between the cellulose molecular chains and the mechanical forces caused by the intertwining of the molecular chains. As the levels of β-CD increased, the storage modulus declined from 1050 Pa to 650 Pa, because the addition of β-CD destroyed the hydrogen bonds and mechanical forces of the cellulose. Moreover, β-CD is difficult to form a gel with high strength with due to its cyclic oligosaccharide and cavity structure. Hence, the presence of β-CD weakened the stability of the three-dimensional network structure of the cellulose-based hydrogel, which demonstrated that cellulose played a supporting role in maintaining its mechanical strength as the backbone of the hydrogel. Figure 5c,d showed the loss modulus (G″) and the phase angle (tanδ) versus the scanning frequency for the CH-g-β-CD hydrogels with different β-CD contents. Independent of the β-CD content and frequency, the loss modulus was far less than the storage modulus (i.e., G′ > G″), and the tanδ was always below 1—suggesting that the elastic behavior of the hydrogel was maintained and that the internal network structure was not destroyed. The Tanδ increased gradually with increases in the β-CD content, which confirmed that the presence of β-CD resulted in the reduced stability of the hydrogel network.

**Figure 6:**
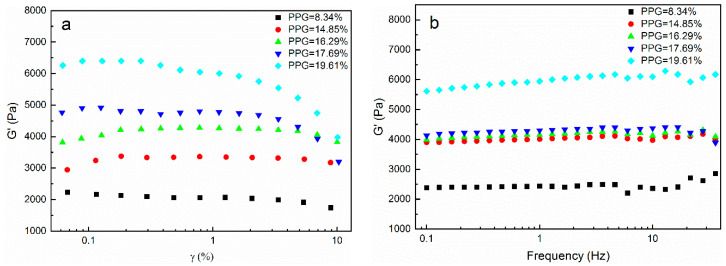

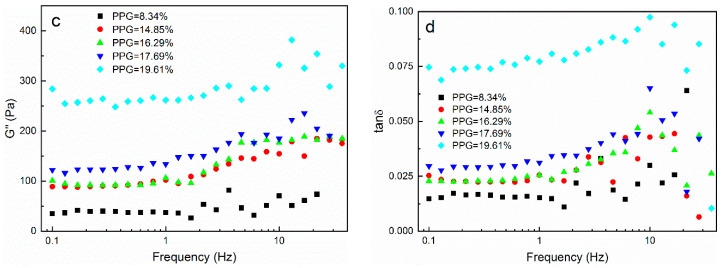
Rheological behavior of the CH-g-β-CD/PPG hydrogels with different PPG contents: (**a**) storage modulus vs. strain; (**b**) storage modulus vs. frequency; (**c**) loss modulus vs. frequency; (**d**) tanδ vs. frequency.

The relationship between the storage modulus and strain amplitude of the CH-g-β-CD/PPG hydrogels with different PPG contents is shown in Figure 6a. All storage modulus curves in Figure 6a remained horizontal and showed strain-independent linear behavior when the strain amplitude was less than 1%, whereas non-linear behavior was exhibited with gradually decreases in G’ as the strain increased further. Consequently, the strain amplitude should be set at 1% during the frequency and temperature scan tests. As illustrated in Figure 6b, the G′ of the CH-g-β-CD/PPG hydrogel was improved from 2500 Pa to 6000 Pa with increases in the PPG content. Compared with the cellulose hydrogel and CH-g-β-CD hydrogel (Figure 5), the G′ of the CH-g-β-CD/PPG hydrogel increased strikingly, which indicated that a more stable three-dimensional network structure was formed in the wake of the enhancement of hydrogen bonding and hydrophobic interactions formed by cyclodextrin–PPG inclusion complexes. The cavity of the β-CD was occupied by the water in the aqueous medium via weak polar–nonpolar interactions, while PPG was substituted into the cavity in the presence of PPG and formed a host–guest complexation. After the water molecules in the cavity were released, the β-CD recombined with the solvated water molecules around the PPG. Desolvation of the PPG and recombination of the solvent molecules contributed to the formation of cyclodextrin–PPG inclusion complexes. As the frequency increased, the storage modulus curves with different PPG contents remained flat—which revealed the good stability of the structure of the supramolecular hydrogel, owing to its intermolecular interactions and the support of the cellulose for the gel network. Like Figure 5b,c, the G″ was far less than the G′ (Figure 6c) and the tanδ was always below 1 (Figure 6d), resulting in the elastic behavior of the CH-g-β-CD/PPG hydrogel. The increasing tanδ demonstrated that the increase in PPG content was conducive to strengthening the stability of the hydrogel network.

**Figure 7:**
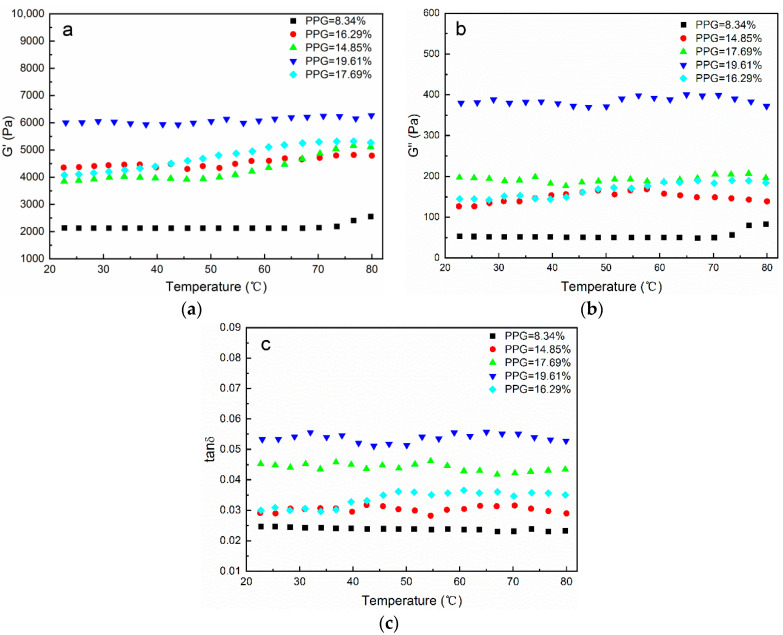
Rheological behavior of the CH-g-β-CD/PPG hydrogels with different PPG contents: (**a**) storage modulus vs. temperature; (**b**) loss modulus vs. temperature; (**c**) tanδ vs. temperature.

The influence of temperature on the G′, G″, and tanδ for the CH-g-β-CD/PPG hydrogels with different PPG contents is shown in Figure 7. The elastic behavior of the supramolecular hydrogel was manifested in that the G″ was far less than the G′ and the tanδ was below 1. As the temperature increased, the curves tended to be horizontal, and the structure of hydrogel network retained excellent stability without drastic changes. The reason for this phenomenon may be that the thermal motion exacerbated by increasing temperatures was restricted in the wake of the hydrogen bonding in the cyclodextrin–PPG inclusion complexes and mechanical forces in the intertwining cellulose.

### 3.5. TGA

**Figure 8:**
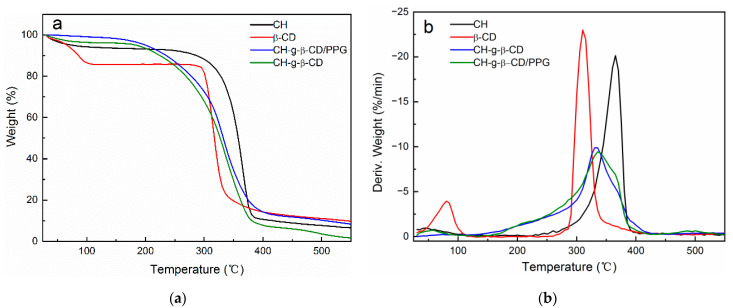
TG-curves (**a**) and DTG-curves (**b**) of the cellulose, β-CD, CH-g-β-CD, and CH-g-β-CD/PPG hydrogel.

It can be seen from Table 1 that the onset temperature of thermal decomposition and the temperature at the maximum weight loss rate (T_max_) were 326 °C and 354.7 °C for the cellulose, respectively. Compared with the cellulose, the thermal stability of the CH-g-β-CD hydrogel receded, with a lower onset temperature and T_max_; this can be attributed to the destruction of the crystalline region of the cellulose after grafting the β-CD and the weaker ether bonds formed between components other than the cellulose [37,38]. The T_max_ of the CH-g-β-CD hydrogel was 328.4 °C, falling in between the cellulose (354.7 °C) and β-CD (316.5 °C). The fact that there was only one thermal decomposition peak for the CH-g-β-CD hydrogel (Figure 8b) is further evidence of covalent bonding between the cellulose and β-CD—as opposed to a physical mixture, where two peaks from the cellulose and β-CD occur simultaneously [39]. The DTG curves showed that β-CD had two thermal decomposition peaks and the peak at 86.5 °C was assigned to the dehydration of the β-CD, while the CH-g-β-CD hydrogel and CH-g-β-CD/PPG hydrogel had no obvious peaks below 200 °C, which revealed the diminished hydration of the β-CD after the grafting and inclusion. There was only one thermal decomposition peak for the CH-g-β-CD/PPG hydrogel, with a T_max_ of 332.8 °C, falling in between the CH-g-β-CD hydrogel (328.4 °C) and PPG (375 °C)—which confirmed the host–guest interactions between the β-CD and PPG in the supramolecular hydrogel [40]. The thermal stability of the CH-g-β-CD/PPG hydrogel was stronger than that of the CH-g-β-CD hydrogel, mainly due to the hydrophobic interactions and hydrogen bonding between the β-CD and inclusive PPG and the aggregation of the PPG chains—resulting in a denser network structure in the supramolecular hydrogel.

### 3.6. Thermosensitivity

**Figure 9:**
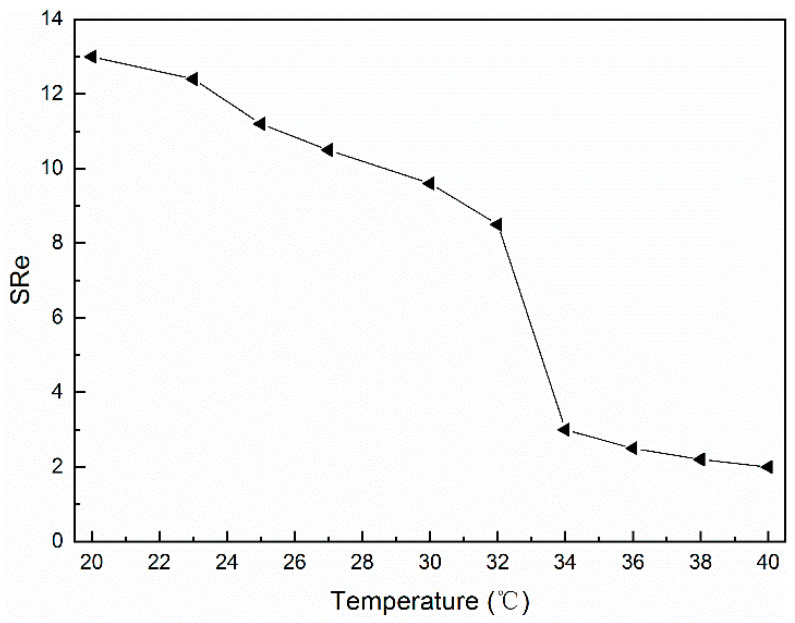
Equilibrium swelling ratio of the CH-g-β-CD/PPG hydrogel as a function of the temperature.

In Figure 9, the equilibrium swelling rate of the CH-g-β-CD/PPG hydrogel tended to decline as the temperature rose, indicating that the resulting supramolecular hydrogel was a negative thermosensitive hydrogel with an LCST of about 34 °C. During the swelling process, water molecules penetrated into the interior of the hydrogel network via intermolecular hydrogen bonds with hydroxyl, causing the interactions between different components to weaken and the hydrogel network to expand in volume. When the hydrogel network expanded and extended, the network structure was subjected to stress and elastic retraction, and the swelling rate of the hydrogel reached equilibrium when these two opposing forces formed a balance [41]. At temperatures below the LCST, the network structure of the supramolecular hydrogel became looser with a high-equilibrium swelling rate due to the penetration of water molecules [42]. On the other hand, when the temperature exceeded the LCST, the hydrogen bonds between the water molecules and the β-CD was broken, the hydrophobic interactions between the β-CD and PPG were further enhanced, and the aggregation of the PPG chains was aggravated. These changes led to the contraction of the hydrogel network and the sharp decline in the equilibrium swelling rate [43].

**Figure 10:**
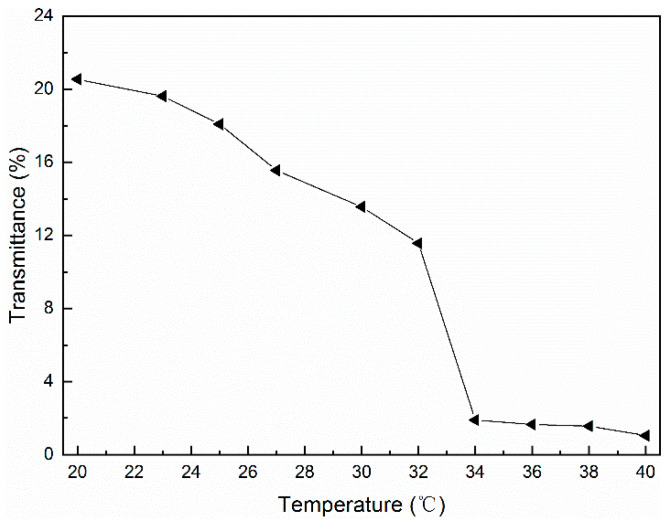
The optical transmittance of the CH-g-β-CD/PPG hydrogel as a function of the temperature.

Figure 10 illustrates that as the temperature increased, the transmittance of the CH-g-β-CD/PPG hydrogel decreased and a transparent opaque phase transition occurred with an LCST of about 34 °C, which was consistent with the results shown in Figure 9. Changes in temperature affected the hydrophobic interactions and hydrogen bonding in the supramolecular hydrogel network and the aggregation of the molecular chains, as well as the volume and transmission of the hydrogel. At temperatures below the LCST, the hydrogen bonding between the water molecules and other components, as well as the good solventization of the PPG, resulted in a loose network structure and high transmittance. In contrast, when the temperature exceeded the LCST, the enhancement of the bonding forces between the various components in the supramolecular hydrogel led to the contraction of the gel, the denseness of the network structure, and a decrease in transmittance.

## 4. Conclusions

The homogeneous grafting of the β-CD on the cellulose molecular chain and the inclusion complexation of the β-CD with the PPG were conducted synchronously under alkaline conditions. Based on the unique amphiphilic structure of cyclodextrin, a cellulose-based supramolecular hydrogel with thermosensitivity was constructed via host–guest complexation and hydrogen bonding. The resulting hydrogel was a negative thermosensitive hydrogel with an LCST of 34 °C. When the temperature exceeded the LCST, the equilibrium swelling rate and light transmittance of the supramolecular hydrogels decreased sharply, implying a sensitive temperature responsiveness. The presence of the amphiphilic structure of the β-CD in the supramolecular hydrogel was an essential reason for its temperature sensitivity. A dense hydrogel network structure with well-developed pores was formed by the interactions between cellulose, β-CD, and PPG. The covalent bonding and non-covalent bonding between the components contributed to the stability of its three-dimensional network structure and thermal stability, with an onset temperature of 294.2 °C. This CH-g-β-CD/PPG supramolecular hydrogel, with superior temperature sensitivity, good thermal stability, and structural stability, has a certain potential application value in smart materials, drug delivery, tissue engineering, and textiles.

## Figures and Tables

**Table 1:** Onset temperature, T_max_, and weight loss during the thermal degradation process of the cellulose, β-CD, and hydrogels.

Sample	Onset Temperature (°C)	T_max_ (°C)	Redidual Mass (%)
cellulose	326	354.7	6.39
β-CD	301.6	316.5	7.75
CH-g-β-CD	284	328.4	1.67
CH-g-β-CD/PPG	294.2	332.8	4.84

## Data Availability

The data presented in this study are available on request from the corresponding author.

## References

[1] Guo W., Li M., Zhou J. (2013). Modeling programmable deformation of self-folding all-polymer structures with temperature-sensitive hydrogels. Smart Mater. Struct..

[2] Ma X., Li Y., Wang W., Ji Q., Xia Y. (2013). Temperature-sensitive poly(N-isopropylacrylamide)/graphene oxide nanocomposite hydrogels by in situ polymerization with improved swelling capability and mechanical behavior. Eur. Polym. J..

[3] Park S.H., Kim R.S., Stiles W.R., Jo M., Zeng L., Rho S., Baek Y., Kim J., Kim M.S., Kang H. (2022). Injectable thermosensitive hydrogels for a sustained release of iron nanochelators. Adv. Sci..

[4] Wu L., Yu Q., Wang S., Mao J., Guo Z., Hu Y. (2022). Synthesis of dual cross-linked ion conductive temperature-sensitive hydrogel and its application in tunable smart window. J. Mater. Sci..

[5] Chen F., Lu G., Yuan H., Li R., Nie J., Zhao Y., Shu X., Zhu X. (2022). Mechanism and regulation of LCST behavior in poly (hydroxypropyl acrylate)-based temperature-sensitive hydrogels. J. Mater. Chem. A.

[6] Bulut E., Turhan Y. (2021). Synthesis and characterization of temperature-sensitive microspheres based on acrylamide grafted hydroxypropyl cellulose and chitosan for the controlled release of amoxicillin trihydrate. Int. J. Biol. Macromol..

[7] Huang H., Qi X., Chen Y., Wu Z. (2019). Thermo-sensitive hydrogels for delivering biotherapeutic molecules: A review. Saudi Pharm. J..

[8] Ge S., Li J., Geng J., Liu S., Xu H., Gu Z. (2021). Adjustable dual temperature-sensitive hydrogel based on a self-assembly cross-linking strategy with highly stretchable and healable properties. Mater. Horiz..

[9] Käfer F., Liu F., Stahlschmidt U., Jérôme V., Freitag R., Karg M., Agarwal S. (2015). LCST and UCST in one: Double thermoresponsive behavior of block copolymers of poly (ethylene glycol) and poly (acrylamide-co-acrylonitrile). Langmuir.

[10] Shi X., Wu J., Wang Z., Song F., Gao W., Liu S. (2020). Synthesis and properties of a temperature-sensitive hydrogel based on physical crosslinking via stereocomplexation of PLLA-PDLA. RSC Adv..

[11] Feki A., Hamdi M., Jaballi I., Zghal S., Nasri M., Amara I.B. (2020). Conception and characterization of a multi-sensitive composite chitosan-red marine alga-polysaccharide hydrogels for insulin controlled-release. Carbohydr. Polym..

[12] Liu S., Liu Z., Wu M., Xu X., Huang F., Zhang L., Liu Y., Shuai Q. (2021). NIR as a “trigger switch” for rapid phase change, on-demand release, and photothermal synergistic antibacterial treatment with chitosan-based temperature-sensitive hydrogel. Int. J. Biol. Macromol..

[13] Xu L., Liang X., You L., Yang Y., Fen G., Gao Y., Cui X. (2021). Temperature-sensitive poly (N-isopropylacrylamide)-chitosan hydrogel for fluorescence sensors in living cells and its antibacterial application. Int. J. Biol. Macromol..

[14] de Oliveira J.P., Bruni G.P., Fabra M.J., da Rosa Zavareze E., López-Rubio A., Martínez-Sanz M. (2019). Development of food packaging bioactive aerogels through the valorization of Gelidium sesquipedale seaweed. Food Hydrocoll..

[15] Batista R.A., Espitia P.J.P., Quintans J.D.S.S., Freitas M.M., Cerqueira M.Â., Teixeira J.A., Cardoso J.C. (2019). Hydrogel as an alternative structure for food packaging systems. Carbohydr. Polym..

[16] Sahiner N., Sagbas S. (2018). Polymeric ionic liquid materials derived from natural source for adsorption purpose. Sep. Purif. Technol..

[17] Gul K., Gan R.Y., Sun C.X., Jiao G., Wu D.T., Li H.B., Kenaan A., Corke H., Fang Y.P. (2022). Recent advances in the structure, synthesis, and applications of natural polymeric hydrogels. Crit. Rev. Food Sci. Nutr..

[18] Cao J., Wu X., Wang L., Shao G., Qin B., Wang Z., Wang T., Fu Y. (2021). A cellulose-based temperature sensitivity molecular imprinted hydrogel for specific recognition and enrichment of paclitaxel. Int. J. Biol. Macromol..

[19] Zhang F., Wu W., Zhang X., Meng X., Tong G., Deng Y. (2016). Temperature-sensitive poly-NIPAm modified cellulose nanofibril cryogel microspheres for controlled drug release. Cellulose.

[20] Crini G. (2014). A history of cyclodextrins. Chem. Rev..

[21] Mura P. (2015). Analytical techniques for characterization of cyclodextrin complexes in the solid state: A review. J. Pharm. Biomed. Anal..

[22] Chaudhary V.B., Patel J.K. (2013). Cyclodextrin inclusion complex to enhance solubility of poorly water soluble drugs: A review. Int. J. Pharm. Sci. Res..

[23] Pandey A. (2021). Cyclodextrin-based nanoparticles for pharmaceutical applications: A review. Environ. Chem. Lett..

[24] Kayaci F., Sen H.S., Durgun E., Uyar T. (2014). Functional electrospun polymeric nanofibers incorporating geraniol–cyclodextrin inclusion complexes: High thermal stability and enhanced durability of geraniol. Food Res. Int..

[25] Harada A., Takashima Y., Nakahata M. (2014). Supramolecular polymeric materials via cyclodextrin–guest interactions. Acc. Chem. Res..

[26] Hu Q.D., Tang G.P., Chu P.K. (2014). Cyclodextrin-based host–guest supramolecular nanoparticles for delivery: From design to applications. Acc. Chem. Res..

[27] Bernardinelli O.D., Lima M.A., Rezende C.A., Polikarpov I., deAzevedo E.R. (2015). Quantitative ^13^C MultiCP solid-state NMR as a tool for evaluation of cellulose crystallinity index measured directly inside sugarcane biomass. Biotechnol. Biofuels.

[28] Wang T., Hong M. (2016). Solid-state NMR investigations of cellulose structure and interactions with matrix polysaccharides in plant primary cell walls. J. Exp. Bot..

[29] Lin N., Dufresne A. (2013). Supramolecular hydrogels from in situ host–guest inclusion between chemically modified cellulose nanocrystals and cyclodextrin. Biomacromolecules.

[30] Mariano M., Bernardinelli O.D., Pires-Oliveira R., Ferreira G.A., Loh W. (2020). Inclusion complexation between α-cyclodextrin and oligo (ethylene glycol) methyl ether methacrylate. ACS Omega.

[31] Abidi N., Cabrales L., Haigler C.H. (2014). Changes in the cell wall and cellulose content of developing cotton fibers investigated by FTIR spectroscopy. Carbohydr. Polym..

[32] Xu F., Yu J., Tesso T., Dowell F., Wang D. (2013). Qualitative and quantitative analysis of lignocellulosic biomass using infrared techniques: A mini-review. Appl. Energy.

[33] Rambabu N., PAnthapulakkal S., Sain M., Dalai A.K. (2016). Production of nanocellulose fibers from pinecone biomass: Evaluation and optimization of chemical and mechanical treatment conditions on mechanical properties of nanocellulose films. Ind. Crops Prod..

[34] Zhao Q., Wang S., Cheng X., Yam R.C., Kong D., Li R.K. (2010). Surface modification of cellulose fiber via supramolecular assembly of biodegradable polyesters by the aid of host-guest inclusion complexation. Biomacromolecules.

[35] Yu S., Yuan J., Shi J., Ruan X., Wang Y., Gao S., Du Y. (2015). One-pot synthesis of water-soluble, β-cyclodextrin-based polyrotaxanes in a homogeneous water system and its use in bio-applications. J. Mater. Chem. B.

[36] Li J., Li X., Ni X., Wang X., Li H., Leong K.W. (2006). Self-assembled supramolecular hydrogels formed by biodegradable PEO–PHB–PEO triblock copolymers and α-cyclodextrin for controlled drug delivery. Biomaterials.

[37] Zhang X., Guo H., Xiao N., Ma X., Liu C., Zhong L., Xiao G. (2022). Preparation and properties of epichlorohydrin-cross-linked chitosan/hydroxyethyl cellulose based CuO nanocomposite films. Cellulose.

[38] Zhang L., Zhou J., Zhang L. (2013). Structure and properties of β-cyclodextrin/cellulose hydrogels prepared in NaOH/urea aqueous solution. Carbohydr. Polym..

[39] Duan J., Jiang J., Han C., Yang J., Liu L., Li J. (2014). The study of intermolecular inclusion in cellulose physical gels. BioResources.

[40] Lin N., Huang J., Dufresne A. (2015). Polysaccharide nanocrystals-based materials for advanced applications. Polysacch.-Based Nanocryst..

[41] Yang K., Wan S., Chen B., Gao W., Chen J., Liu M., He B., Wu H. (2016). Dual pH and temperature responsive hydrogels based on β-cyclodextrin derivatives for atorvastatin delivery. Carbohydr. Polym..

[42] Singh N.K., Lee D.S. (2014). In situ gelling pH-and temperature-sensitive biodegradable block copolymer hydrogels for drug delivery. J. Control. Release.

[43] Zheng S., Hu J., Xu X., Chen X., Shen D. (2014). Thermo-and pH-sensitive hydrogels containing the β-cyclodextrin moiety for controlled protein release. Monatsh. Chem..

